# Hereditary Neuroses in Children.—III

**Published:** 1897-05-29

**Authors:** 


### Modern Sociology.

### HEREDITARY NEUROSES IN CHILDREN.—III.

Neurotic children are not unfrequently subject to "night terrors" (*pavor nocturnus*), which may be regarded as a form of convulsive affection, the psychical centres having in such cases a tendency to explosive action during sleep. Sometimes, as in a case reported by Henoch, in which a girl of 10 first had fits and subsequently, on their cessation, night terrors, they bear a definite relation to epilepsy. The children of "highly-strung" parents are specially liable to such attacks, which indeed may be regarded as a manifestation of inherited neurosis.

Laryngeal spasm (*laryngismus stridulus*) is another affection to which children of neurotic stock are specially prone, and it is more common with those of rickety constitution. It sometimes alternates with eclampsia, and is not often met with beyond the period of dentition, though with mentally-defective children of convulsive tendency it is sometimes observed as late as the seventh or eighth year. Spasm affects, also, in nervous children not only the laryngeal muscles, but those of respiration; and the condition of spasmodic asthma is occasionally observed in early youth where there is strongly-marked neurotic inheritance. An asthmatic tendency is noted as a factor in the family history of mentally defective children.

The irregular discharge of nervous energy may take the form of a passionate outburst instead of a convulsion. What Marshall Hall* has designated "temper disease" is sometimes seen in children of good mental ability and quick wit, who labour under an inherited predisposition to neurosis. The perversities of such cases are most trying to parents themselves neurotic, and more judicious discipline is usually possible in the hands of strangers than in the family circle. Children of this type who are mismanaged are apt, as they grow up, to pass into the stage of adolescent insanity. Insanity, as distinguished from imbecility, is not common in early childhood; but when it exists, it is invariably (as Dr. Clouston tells us†) "in the children of very neurotic or insane parents or grandparents." He instances the case of a "small, melancholic child of six, who wept and wailed, and was sad about imaginary things, such as going to hell, and having taken a drink of water when it was wrong to do so." He refers also to two cases of suicidal melancholia occurring from seven to ten years of age. Transient acute mania is exceptionally met with in young children of neurotic antecedents. Of late years, child suicide has been increasing in England and in most Continental countries; and, as Dr. Strahan points out,‡ "it is significant of the increase of this most pitiful form of self-destruction that the Registrar-General has latterly found it convenient to add an extra column for the reception of self-destroyers whose ages are between five and ten years"! Recently the number of suicides of children under 15 years of age has averaged over 10 annually, in England and Wales alone. It has been remarked that educational strain is probably a factor in the increase of juvenile suicidal insanity, which is most common in countries in which education is forced on most strongly.

* "Observations in Medicine." Dr. M. Hall. First series, p. 82.

† "Neuroses of Development," p. 113.

‡ "Suicide and Insanity," p. 175. (London, 1893.

When the inherited neurosis is intense, there may often be traced from early infancy a perverted state of feeling (described by the Germans as *Primäre Verrücktheit*), in which "envy, hatred, malice, and all uncharitableness" are prominent characteristics. The child may be intellectually bright, but there are lurking suspicions, dismal forebodings, and sometimes overweening self-consciousness, which detract from the freshness and innocent enjoyment of childhood, and, as age advances, are apt to crystallise into actual delusions.

Akin to these cases, though differing in some characteristics, are those which have been classed under the designation of "moral imbecility" in childhood. The moral imbecile is described by Dr. Shuttleworth* as "the despair of his parents, the *bête noir* of the institution, the perplexing puzzle of the jurist," and he is referred to as "the ill-fated product of inherited nervous instability and ancestral criminal instincts." An instance is given of "three nice-looking children, sisters and brother, who at times would appear models of propriety, while at others they had all the characteristics of little demons. With innocent expression they would furtively accomplish the most abominable mischief, and after meekly acknowledging the error of their ways, would emphasise their apology by a missile flung at the head of the person who had attempted to bring them to repentance!" Children of this type have sometimes to be excluded from school (for which they are intellectually fit) in consequence of filthy habits, which seem to be ingrained into their nature. A paroxysmal tendency to wrong-doing has been traced in some cases, and has been attributed to a species of "psychical epilepsy."

Various hysterical affections are found in children of neurotic predisposition, often at a very early age. There is a type of feeble-minded children of nervous temperament, very apt (from lack of inhibitory power) to break down into what may be called hysterical paroxysms either of laughing or crying — often indifferently—from causes inadequate to produce such excitement in normal children. Henoch recounts several cases in which the mimetic muscular affections of hysteria were seen in children about ten years of age, in some resembling the symptoms of hystero-epilepsy, and in others those of hysterical paralysis.

In hysterical cases occurring in children there is no doubt a "pathologically premature development of the emotional brain-centres" † due to bad heredity; and this is sometimes accompanied by a premature sexual development. As a consequence, precocious erotic tendencies are occasionally met with in such children; and evil practices must be rigorously guarded against, even from a very early age.

The changes incident to the period of puberty bring about, in those predisposed by inheritance, developmental neuroses of great significance and far-reaching consequences; but the consideration of these we must leave for our next article.

* "Mentally-deficient Children," p. 99.

† Clouston, op. cit., p. 113.

